# Rapidly progressing subperiosteal orbital abscess: an unexpected complication of a group-A streptococcal pharyngitis in a healthy young patient

**DOI:** 10.1186/1746-160X-8-28

**Published:** 2012-10-16

**Authors:** Fulvia Costantinides, Roberto Luzzati, Daniele Tognetto, Gabriele Bazzocchi, Matteo Biasotto, Gian Carlo Tirelli

**Affiliations:** 1 Division of Oral Medicine, Department of Dental Sciences, Piazza dell’Ospitale 1, University of Trieste, Trieste 34100, Italy; 2 Department of Infectious Diseases, Piazza dell’Ospitale 1, University of Trieste, Trieste 34100, Italy; 3 Department of Ophthalmology, Piazza dell’Ospitale 1, University of Trieste, Trieste 34100, Italy; 4 Department of Radiology, Piazza dell’Ospitale 1, University of Trieste, Trieste 34100, Italy; 5 Department of Head and Neck Surgical Sciences, Strada di Fiume 447, University of Trieste, Trieste 34149, Italy

**Keywords:** Group-A streptococcal pharyngitis, Orbital abscess, Functional endoscopic sinus surgery, Visual acuity, Dental examination

## Abstract

**Introduction:**

Complications associated to group-A streptococcal pharyingitis include non-suppurative complications such as acute rheumatic fever and glomerulonephritis and suppurative complications such as peritonsillar or retropharyngeal abscess, sinusitis, mastoiditis, otitis media, meningitis, brain abscess, or thrombosis of the intracranial venous sinuses.

**Case presentation:**

We described a case of a 15-year-old patient with a history of acute pharyngodinia early followed by improvise fever and a progressive formation of a diffuse orbital edema, corneal hyperaemia, diplopia and severe decrease of visual acuity.

The patient was surgically treated with functional endoscopic sinus surgery (FESS) after the response of a maxillofacial computed tomography scans that showed a pansinusitis complicated by a left orbital cellulites. Numerous colonies of *Streptococcus pyogenes* were found in the samples of pus and an antibiotic therapy with meropenem was initiated on the basis of the sensitivity test to antibiotics. The patient was finally discharged with diagnosis of left orbital cellulites with periorbital abscess, endophtalmitis and acute pansinusitis as a consequence of streptococcal pharyngitis.

**Conclusion:**

The case highlights the possible unusual complication of a group-A streptococcal pharyingitis in a immunocompetent child and the needing of a prompt surgical and medical approach toward the maxillofacial complications associated to the infection.

## Introduction

Group A beta-haemolitic streptococcus (GAS) being the most common etiology of sore throats caused by bacteria. It has been estimated that GAS is responsible for around 15-30% of cases of acute pharyngitis in children [1]. Streptococcal pharyngitis is most common in children 5 to 12 and presents with a predominant sore throat and a temperature higher than 38.5°C. Symptoms include fever, chills, myalgias, headaches and nausea. Physical findings may include pharyngeal and tonsillar erithema and exudates and cervical adenopathy [2]. Sequelae associated to the GAS infection include non-suppurative (or post-streptococcal) complications as rheumatic fever and glomerulonephritis and suppurative complications as cervical lymphadenitis, peritonsillar or retropharyngeal abscess, sinusitis, mastoiditis, otitis media, meningitis, thrombosis of the intracranial venous sinuses, endocarditis, pneumonia, sepsis. In rare cases, necrotizing fascitis, myositis and streptococcal toxic shock syndrome have been described [3].

We report the case of a previously healthy 15-year-old girl affected by pansinusitis and subperiosteal orbital abscess following an acute GAS pharyngitis and successfully treated with functional endoscopic sinus surgery (FESS).

### Report of a case

A 15-year-old girl was admitted to the Department of Ophtalmology (University of Trieste) for high-grade fever (body temperature 40.2°C), severe decrease of visual acuity, left eyelid edema and hyperaemia. Five days before, she had started to complain pharyngodinia, odynophagia, and fever treated with antipyretics by the general physician. Symptoms were followed by progressive pain at the medial left orbital canthus and headache. Her past medical history was unremarkable, and no previous visual compromise was reported. At admission, clinical ophthalmological examination demonstrated a severe edema and erythema of the eyelids with a slight proptosis associated with a conjunctival chemosis and hyperaemia. An ophthalmoplegia was present and the patient referred diplopia and pain on eye movements. Visual acuity was 20\20 in right eye and 20\50 in left eye. In addition, a pharyngeal diffuse erythema with a bilateral anterior cervical lymphadenomegaly was present. A pharmacological therapy with acetaminophen 500 mg per os (when fever was higher than 38°C) and cefaclor 500 mg per os every six hours was administered. Laboratory findings showed normal value of the white cell blood count (9.01 × 10^3^/mm^3^) with moderate neutrophilia (77%). S-C-reactive protein (CRP) and erythrosedimentation rate were increased with values of 142.8 mg/L (normal value < 5 mg/L) and 92 mm/h (normal value < 10 mm/h), respectively.

**Figure 1:**
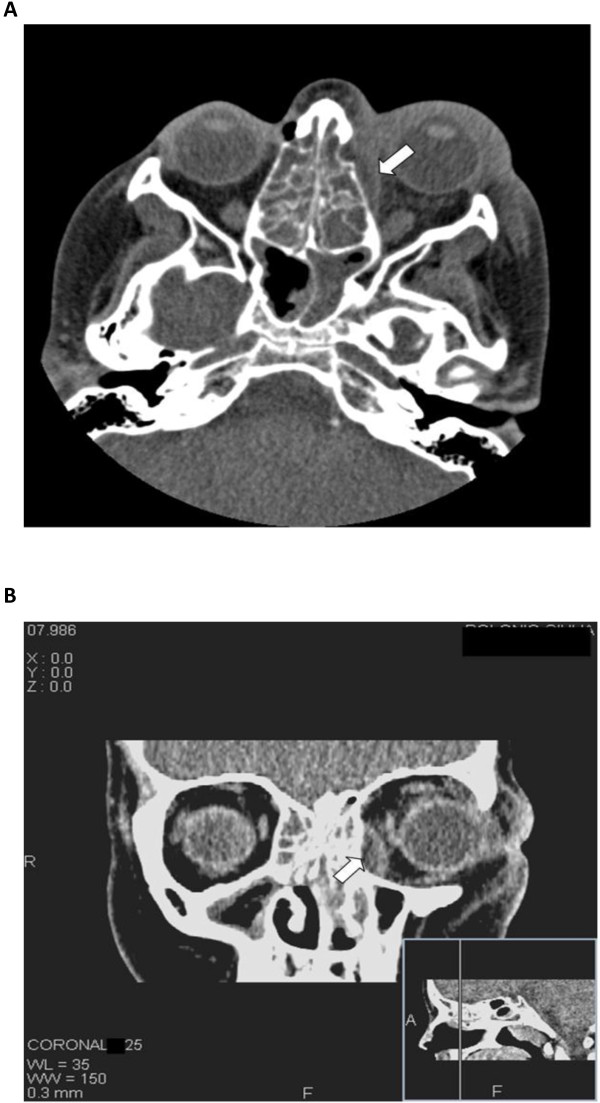
**A-B**. **Axial** (**A**) **and coronal **(**B**) **CT unenhanced scan through the middle orbit. **The images show: increased thickness of skin and soft tissue overlying the medio-lateral area of the left orbit, decreased transparency of the ipsilateral intraconal and extraconal fat tissue and a subperiosteal abscess along the medial wall of the left orbit (white arrows), adjacent to the opacified ethmoid air cells with resultant lateral displacement of the medial rectus muscle (b) A partial obliteration of sphenoid (**A**) and maxillary sinuses (**B**) is also visualized.

CT scans showed an increased thickness of skin and soft tissue overlying the medio-lateral area of the left orbit with a decreased transparency of the fat tissue. At the anteromedial ethmoidal cell area, a circumscribed liquid collection with a convex border directed toward the internal orbit was appreciable. The liquid mass caused a slight deviation of the medial rectum muscle that appeared thickened but regular in morphology. A complete obliteration of all the paranasal cavities with little hydro-air levels in both maxillary sinuses and thinning of the ethmoidal bone plates were observed with a possible bilateral interruption of the papyracea lamina (Figure 1A-B). After three hours from the admission, the patient was transferred to the Department of Head and Neck Surgical Sciences (University of Trieste) to undergo functional endoscopic sinus surgery (FESS) under general anaesthesia. An extensive osteoplastic procedure of the natural ostium of the left maxillary sinus was performed. During the anteroposterior ethmoidectomy and revision of the left anterior ethmoidal cells, near the nasolacrimal duct, the papyracea lamina was surgically interrupted for a short trait and a large purulent collection was evacuated from the orbita. The latter finding was consisting with a subperiosteal abscess. Samples of purulent fluid from the orbital area and maxillary sinus were collected for microbiological culture, and samples of mucosal tissues were sent for the hystopathologic examination. The patient was given an antibiotic therapy with meropenem 1g three times daily intravenously as indicated by the specialist in infectious diseases. The same day of the surgery, the temperature dropped at 36.6°C. At day 1 post-surgery the patient was afebrile, the ophthalmologic clinical status was improving, and CRP decreased to 112.6 mg/L while erythrosedimentation rate remained unvaried (93 mm/h).

**Figure 2:**
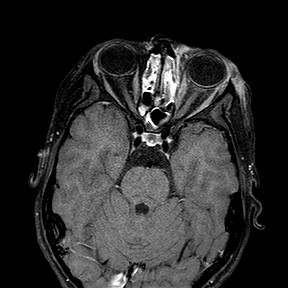
**Axial contrast**-**enhanced fat suppressed T1**-**weighted MR.** The image demonstrates ethmoid and sphenoid sinusitis and an heterogeneous enhancement of left eyelid tissues and of orbital fat. The left orbital extraocular muscles show a moderate enhancement due to edema and hyperaemia. Left medial rectus muscle is also moderately thickened.

**Figure 3:**
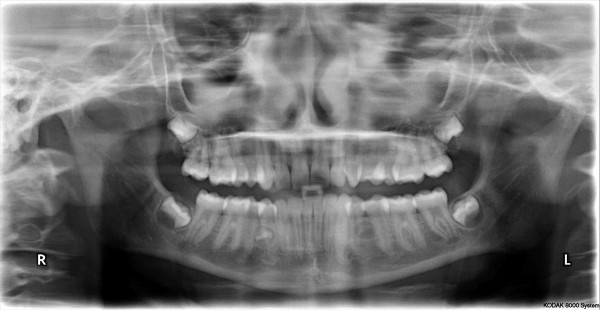
**Orthopantomography. **Image shows a bilateral opacity of the maxillary sinuses.

A cerebral angio-magnetic resonance imaging (A-MRI) did not show any intracranial lesion including the left eyeball and the ophthalmic nerve. A diffuse signal alteration was present in the left retro-bulbar adipose tissue associated to a moderate edema and hyperaemia of the extrinsic musculature (Figure 2). A dental examination was performed to evaluate the possible odontogenic origin of the infection. The patient was not affected by dental or periodontal disease although the vertical percussion of posterior maxillary teeth and the bilateral palpation of the vestibular maxillary region exacerbate a localized pain (VAS= 4). An orthopantomography showed a complete bilateral opacity of the maxillary sinuses but no periapical lesions or any dental involvement (Figure 3).

In the second day after surgery the patient had no fever (36.5°C) and the pharingodinia decreased. Histology of the mucosal samples showed connective tissue characterized by diffuse and conspicuous acute and chronic inflammatory infiltrate. Culture of the orbital and ethmoidal essudates showed numerous colonies of group-A *Streptococcus pyogenes* which was sensitive to penicillin and cephalosporin derivates, to macrolides, tetracycline, chinupristine/dalfopristine, vancomycine and clindamycin. An intermediate sensibility was observed for levofloxacine. At day 7 post-surgery, the patient remained afebrile, her left orbital swelling, proptosis and diplopia were disappearing and her visual acuity in her left eye rose to 20\20. The patient was discharged on antibiotic therapy with amoxicillin plus clavulanic acid 1g three times daily, orally for 5 day. After 6 months post-hospital admission, the clinical follow-up of our patient was completely favourable.

## Discussion

The orbital septum divides the preseptal space (soft tissues of the eyelid) from the orbital space (postseptal space) so that periorbital or preseptal cellulitis involve only the lids and not the orbit, whereas orbital or postseptal cellulitis is much more uncommon and involves the soft tissues of the bony orbit. An opthalmological examination is mandatory in assessing proptosis, chemosis, opthalmoplegia or decreased visual acuity as these findings highlight the presence of postseptal orbital cellulitis. However, the distinction beteween preseptal cellulitis and orbital involvement cannot be made with clinical examination alone and delay in treatment can result in blindness in up to 10% of patients [4]. Orbital cellulitis is a serious infection in children and can result in significant complications as blindness, cavernous sinus thrombosis, meninigitis, subdural empyema and brain abscess [5]. In the preantibiotic era, 20% of patients with orbital cellulitis had permanent loss of vision and 17% died for central nervous system complications, today these percentages decreased but have not still been eliminated (15 to 30% of patients develop visual sequelae) [6].

Orbital complication accounts for 74 - 85% of complications arising from acute sinusitis and usually this is secondary to acute ethmoidal sinusitis since the ethmoid sinus is separate from the orbit only by the papyracea lamina [7]. In a paediatric series, Nageswaran et al. found that 98% and 71% of their patients with orbital cellulitis were affected by ethmoid or maxillary sinusitis respectively [5]. Furthermore bilateral pansinusistis is the most common presentation [6]. As in this case, an abscess may be present in the subperiostium of the lateral wall of the lamina papyracea [8]. It was estimated that the incidence of a subperiosteal abscess in orbital infections is about 15% in children [6]. The etiology of orbital cellulitis is usually unknown because blood cultures are often negative. Sinus cultures reveal typical acute sinusitis pathogens including *Streptococcus pneumoniae*, *Haemophylus influenzae*, *Moraxella catharralis*, *Streptococcus pyogens*, *Staphylococcus aureus*, *α*- and nonhemolytic streptococci and anaerobic bacteria of the upper respiratory tract [5]. Subperiosteal abscess cultures showed *S*. *pneumoniae*, group A streptococci, *H*. *influenzae*, as the major pathogens in a previous paediatric series [9]. Polymicrobial infections are also common and may be more frequent in older versus younger children [10].

The management of an “acute orbit” depends on the cause and severity of the infection. All patients affected by orbital cellulitis should be treated with intravenous antibiotics whereas they should undergo surgical drainage of abscesses and involved sinuses only in presence of large abscess, complete ophtalmoplegia or significant visual impairment, as in the our patient [8]. Maxillo-facial CT scan is indicated to evaluate the extension of the infection and to identify children who are most likely to benefit from surgical intervention [9]. Because of the aggressive nature of a subperiosteal orbital abscess, we agree with Rahbar et al. in obtaining a CT scan even if the only presentation is preseptal cellulitis [6]. In our case, CT images showed a massive involvement of the paranasal cavities including the ethmoidal cells and the subperiosteal abscess of left orbit. Impairment of vision, periorbital erythema and hyperemia, proptosis, together with radiological findings, indicated an immediate surgical approach to avoid the potential loss of vision and the devastating morbidity associated to the subperiosteal orbital abscess [6]. The surgical drainage of the abscess was afforded with FESS. This transnasal endoscopic technique provided a quick and safe drainage of the paranasal sinuses, orbita, anterior skull base avoiding facial scars as well as hastening the post-operative recovery period [11,12]. Rahbar et al. found that orbital subperiosteal abscess in children can be successfully and safely managed by a transnasal endoscopic approach in selected patients [6]. The choice of method of surgical drainage should be based on the location of the abscess and the experience of the surgeon. Medial and inferior orbital abscesses can be treated with an endoscopic approach while superior localization generally requests an external drainage.

After the emergency surgical treatment, a polispecialistic examination was immediately requested to choose the best antibiotic option, to examinate the dental status, and to exclude intracranial spreading of the infection. Considering this last complication, intravenous therapy with meropenem, a broad-spectrum antibacterial agent of the carbapenem family, was chosen. Meropenem is indicated as empirical therapy prior to the identification of causative organisms, or for disease caused by single or multiple susceptible bacteria in both adults and children with a broad range of serious infections [13].

The dental status was evaluated since the odontogenic nature of the sinusal and orbital infection has to be excluded, especially when the localization is monolateral. Finally, the intracranial spreading of the infection was excluded by A-MRI.

The result of the microbiological analysis (positive for GAS) led to consider a strong correlation between the pharyngitis and the sinusal/orbital involvement and to discharged the patient with the final diagnosis of left orbital cellulitis with periorbital abscess, endophtalmitis and acute pansinusitis as a consequence of GAS pharyngitis.

Group A streptococcal pharyngitis is usually a self-limited disease, and therapy can generally be safely postponed for up to 9 days after the onset of symptoms to prevent the occurrence of major nonsuppurative sequelae. However, according to guidelines of the Infectious Diseases Society of America, early initiation of antibiotic therapy results in faster resolution of signs and symptoms [14]. Furthermore a Cochrane review of randomized, placebo-controlled trials showed that antibiotic therapy significantly reduces the risk of acute otitis media (relative risk 0.30; 95% CI, 0,15-0.58) and peritonsillar abscess (relative risk 0.15; 95% CI, 0.05-0.47) [15]. Thus, antimicrobial therapy is recommended for subjects with symptomatic pharyngitis in the presence of GAS in the throat confirmed by culture or RADT [16]. Clinical scoring systems have been developed to predict the likelihood of streptococcal infection among children and adults with sore throat [17]. Presence of fever (temperature > 38°C), absence of cough, tonsillar swelling or essudate, tender and enlarged anterior cervical lymphnodes are correlated to approximately 30 to 50% probability of positive results of a throat culture or RADT. In this case, despite the clinical presentation, the streptococcal infection was not previously diagnosed. However, the serious complications seen in the patients are usually unexpected in a 15-years immunocompetent girl although it has been recently observed an increase in the incidence of head and neck infections especially associated with acute sinusitis due to group A streptococcal infections in children [18]. This trend may reflect an increase of virulence related to the evolving biology of streptococcal organism and can justify the onset of aggressive and rapidly progressive infections also in previously health children. Much of the increase in invasive dissemination of *Streptococcus Pyogenes* (noted in last 15 years) has been associated with M protein types M1 and M3 that prevent phagocytosis of the bacteria by inhibiting the interaction with complement [18].

## Conclusion

In conclusion, our case focused the attention on:

– the possible spreading of a streptococcal pharyngeal infection towards the orbital involvement that may require a multispecialty emergency approach;

– the need of a prompt diagnosis and therapy for streptococcal pharyngitis that might prevent such potentially sight- and life-threatening complication;

– the surgical management of subperiosteal orbital abscess that can by safely performed with FESS in young patients affected by of a subperiosteal abscess as a consequence of a streptococcal pharyngeal infection.

### Consent

Written informed consent was obtained from the parents of the patient for publication of this case report and any accompanying images. A copy of the written consent is available for reviewer by the Editor-in-Chief of this journal.

## Abbreviations

GAS: Group A beta-haemolytic *Streptococcus*; RADT: Rapid antigen detection test; FESS: Functional endoscopic sinus surgery; CT: Computerized tomography; A-MRI: Angio-Magnetic Resonance Imaging.

## Competing interests

The authors declare that they have no competing interests.

## Authors’ contributions

FC drafted the manuscript and participated in the management of the case (oral and maxillofacial examination). RL, DT, GB, MB and GT participated in management of the case (respectively treating: RL the diagnosis and antibiotic therapy, DT the ophthalmic assessment and re-evaluation, GB the radiological investigation, MB the oral and maxillofacial examination, GT the surgical therapy) and in drafting the manuscript revising it critically. All authors gave final approval of the version to be published.
